# Comparison of menstrual blood and endometrial biopsy as specimens in the diagnosis of female genital tuberculosis: a systematic review

**DOI:** 10.1186/s12879-026-12564-8

**Published:** 2026-01-29

**Authors:** Usha Nayak, Tejaswini Baral, Sonal Sekhar Miraj, Mohan K Manu, Muralidhar D Varma, Sandeep Samethadka Nayak, Jyothsna Manikkath

**Affiliations:** 1 Department of Pharmacy Practice, Manipal College of Pharmaceutical Sciences, Manipal Academy of Higher Education, Manipal, Karnataka State 576104 India; 2 Department of Respiratory Medicine, Kasturba Medical College, Manipal, Manipal Academy of Higher Education, Manipal, Karnataka State 576104 India; 3 Department of Infectious Diseases, Kasturba Medical College, Manipal, Manipal Academy of Higher Education, Manipal, Karnataka State 576104 India; 4 Department of Internal Medicine, Yale New Haven Health Bridgeport Hospital, 267 Grant Street, Bridgeport, CT 06610 USA; 5 Department of Pharmaceutics, Manipal College of Pharmaceutical Sciences, Manipal Academy of Higher Education, Manipal , Karnataka State 576104 India

**Keywords:** Female genital tuberculosis, Menstrual blood, Diagnosis, Non-invasive, Sensitivity, Specificity

## Abstract

**Background:**

Female genital tuberculosis (FGTB) continues to be a significant problem worldwide. Endometrial biopsy (EB) is the most effective sample for detecting FGTB. However, biopsy is invasive and causes unwarranted suffering. Furthermore, a few studies have investigated non-invasively obtained menstrual blood (MB) as an alternative diagnostic sample for detecting FGTB. Hence, in this study, we are assessing MB as an alternative sample to EB for diagnosing FGTB.

**Method:**

A systematic literature search was conducted using the electronic databases PubMed, Scopus, Web of Science, and Embase until September 2024. All original studies that compared MB with samples of EB for diagnosing FGTB were included.

**Results:**

A total of nine studies were obtained. The sensitivity of tests using MB as a sample ranged from 33.3% to 91.7%, while the specificity ranged from 82.9% to 97.3%. In the EB group, the sensitivity ranged from 64.8% to 95.8%, and the specificity ranged from 84.3% to 97.5%.

**Conclusion:**

The review suggests that MB could be a potential sample for diagnosing FGTB, as the specificity of tests using this sample is comparable to that from the EB group.

**PROSPERO registration:**

The study protocol was registered in the International Prospective Register of Systematic Reviews (PROSPERO) with the identification number CRD42024509199.

**Supplementary Information:**

The online version contains supplementary material available at 10.1186/s12879-026-12564-8.

## Background

Female genital Tuberculosis (FGTB) is one of the frequently observed forms of extrapulmonary tuberculosis (EPTB) [1]. FGTB occurs when *Mycobacterium tuberculosis* infects the female genital organs, including the fallopian tubes, endometrium, ovaries, vagina, and cervix. The fallopian tube is the most affected part [2]. In India, the rates of FGTB vary from 16.1% to 19% [3]. Reports on the prevalence vary, with 45.1 cases per 100,000 women in a community-based study in the Andaman Islands [4] and 48.5% among infertile women in Northern India [5]. FGTB can occur in females of any age group; however, reproductive age, i.e. 15 to 49 years old, are the most vulnerable [2, 6]. The commonly observed symptoms of FGTB are abdominal pain, amenorrhea, post-menopausal bleeding, menorrhagia, intermenstrual bleeding, infertility, and dyspareunia [7]. Among these, infertility is the most prevalent symptom and consequence, observed in approximately 70.7% of women with FGTB. The prevalence of primary and secondary infertility in FGTB is 75.7% and 24.3%, respectively [8].

Early diagnosis of FGTB is associated with better chances of spontaneous conception [9] and lowered chances of developing multi-drug resistant (MDR) TB [10]. Endometrial biopsy (EB) obtained through curettage or aspiration during the luteal phase, is the preferred sample for diagnosing FGTB using polymerase chain reaction (PCR) [11–13]. However, EB collection is invasive, involving removal of tissue from the uterine lining via a catheter through the cervix, which can cause pain and anxiety [14]. Along with this, it also leads to abdominal cramps and severe infections post-procedure [15]. Hence, identifying a reliable non-invasive sample is essential.

MB is a sample that is easy to obtain via non-invasive techniques [16, 17]. Globally around 1.8 billion women menstruate every month [18], where the menstruating phase lasts anywhere between 3 and 7 days. This blood can be easily collected on sanitary pads, tampons, or menstrual cups [19, 20], making MB a readily available sample at a significantly lower cost than EB samples. There exists a certain amount of ambiguity in whether MB is sensitive and specific enough to diagnose FGTB. Therefore, we conducted a systematic review to determine the diagnostic potential of MB in comparison to EB for FGTB.

## Methods

### Registration and protocol

The systematic review protocol was registered on the International Prospective Register of Systematic Reviews (PROSPERO), with the identification number CRD42024509199. The review was conducted in accordance with the Preferred Reporting Items for Systematic Review and Meta-Analysis (PRISMA) 2020 guidelines.

### Review objectives

The research question for the systematic review is “Is menstrual blood a suitable specimen compared to endometrial biopsy for the detection and diagnosis of female genital tuberculosis ?” The research question was divided into ‘P’, ‘I’, ‘C’, and ‘O’ (population, intervention, comparison, and outcome) formats. The defined ‘population’ was female without age and ethnicity restrictions. ‘Intervention’ was MB sample in diagnosis of FGTB. The ‘comparator’ was EB sample in diagnosis of FGTB. The ‘outcome’ measure was sensitivity and specificity.

### Search strategy

A systematic literature search was carried out to select relevant studies in the electronic databases from inception till September 2024. Advanced search was performed on PubMed, Scopus, Web of Science, and Embase. Database searches used key terms and MeSH headings such as ‘menstrual blood,’ ‘female genital tuberculosis,’ and ‘Mycobacterium tuberculosis,’ combined with Boolean operators without publication date and language restrictions. (Supplementary file 2)

### Article selection eligibility criteria

All original research studies that compared MB with EB for diagnosing FGTB were included. Review articles, letters to editors, conference proceedings, and abstract-only papers were excluded.

### Article screening and data extraction process

Articles were selected and screened by title and abstract, followed by full-text screening based on our predefined eligibility criteria. Two independent reviewers (U.N. and T.B.) performed the quality assessment, and any disagreements were settled through consensus or discussion with another reviewer (J.M.). A pre-designed data extraction sheet was used to extract data from the incorporated studies. The following variables were extracted: author name, title, year of publication, digital object identifier, study design, place of study, inclusion and exclusion criteria, number of patients in the study group, patient demographics, sample used for diagnosis, and diagnostic technique used (Supplementary file 1).

### Quality assessment

Quality Assessment of Diagnostic Accuracy Studies-2 (QUADAS-2) risk of bias assessment scale was used to assess the methodological quality of the included studies. Two independent reviewers (U.N. and T.B.) performed the quality assessment, and any disagreements were settled through consensus or discussion with another reviewer (J.M.).

## Results

### Study selection

**Fig. 1:**
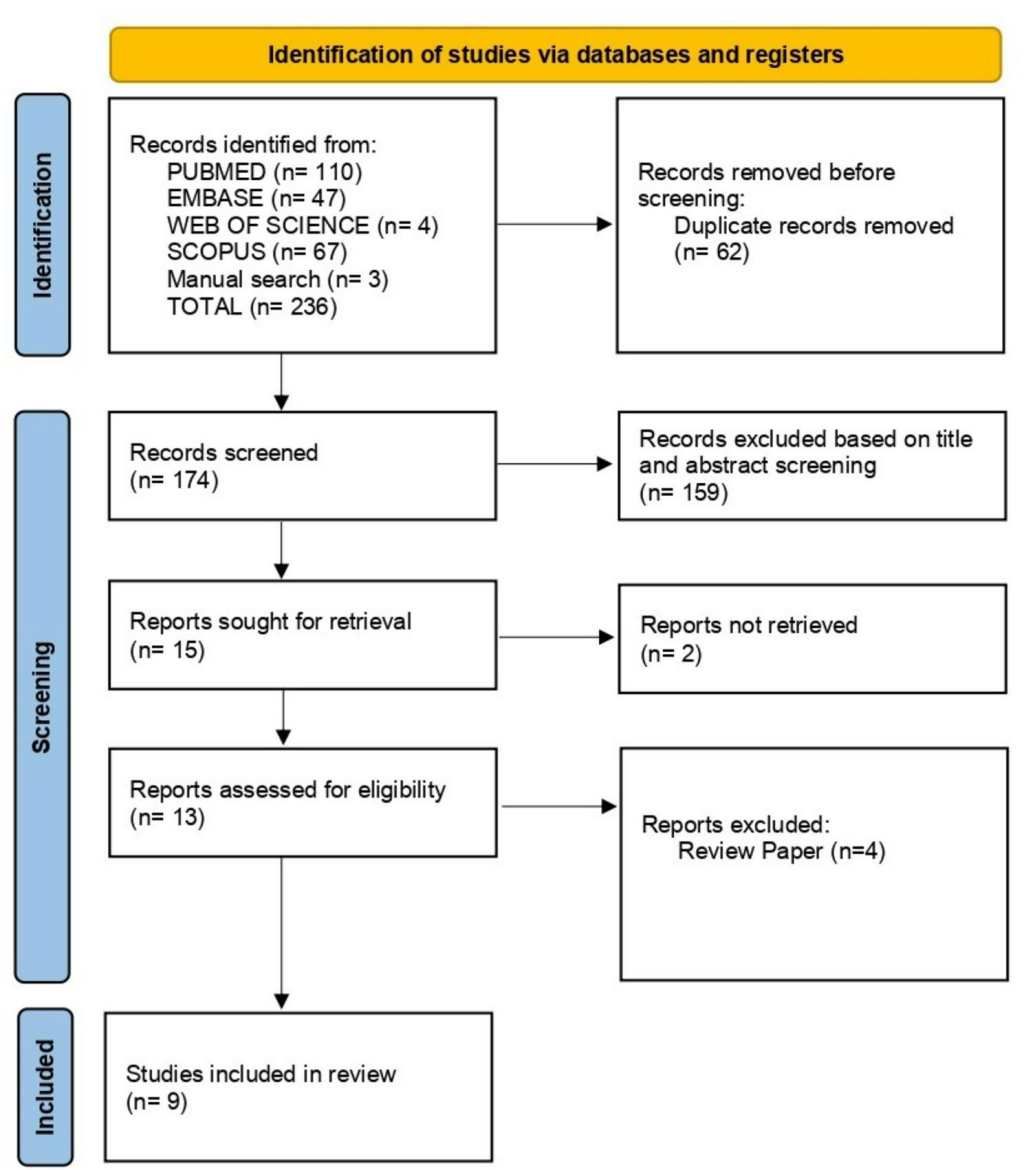
PRISMA 2020 flow chart of the screening and selection process

A total of 236 records were obtained, out of which 233 were from the above-mentioned databases, and 3 were found following a manual literature reference search. Out of the 236 records, 62 duplicate records were removed before the screening. After removing duplicates, 174 records were screened based on title and abstract. Of these, 159 records were excluded as they were unrelated to the scope of the review. Two of the 15 records were not available for the full-text screening. Therefore, 13 records were assessed for eligibility based on the study criteria. From these, 4 records were excluded since they were review articles. After full-text screening, 9 papers were finalised for our review [16, 21–28]. The PRISMA flow chart of article selection is illustrated in Fig. 1.

### Study characteristics

A total of 9 studies were obtained [16, 21–28]. Of these, 7 were cross-sectional, and one was a prospective study [28], and one was a retrospective study [16]. All studies were from India except for one cross-sectional study from Turkey [22]. Data from these studies was classified into MB and EB groups (Table 1).

**Table 1:** Basic characteristics of included studies

S. N	Study (ref)	Study design	MB		EB	
			Diagnostic test	Sample size	Diagnostic test	Sample size
1	Kamra et al., 2022 [27]	Cross-sectional study	M- nested PCR	3	M- nested PCR	22
			M- PCR	3	M- PCR	22
2	Chaubey et al., 2019 [26]	Cross-sectional study	Nested PCR	194	PCR nested	194
3	Paine et al., 2018 [23]	Cross-sectional study	Multiplex PCR	195	PCR	195
					Histopathology	195
4	Sindhoora et al., 2016 [28]	Prospective study	Real-time PCR	50	PCR	50
5	Patil et al., 2015 [24]	Cross-sectional study	Versa TREK culture	123	Versa TREK culture	42
			MTD	123	MTD	42
6	Malhotra et al., 2012 [25]	Cross-sectional study	Real-time PCR	17	Real-time PCR	524
					AFB smear test	524
					Culture positivity test	524
7	Kashyap et al., 2012 [16]	Retrospective study	Smear microscopy	21	Smear microscopy	1226
			Culture positivity test	21	Culture positivity test	1226
8	Aka et al., 1997 [22]	Cross-sectional study	Culture positivity test	57	Histopathology	57
9	Sharma et al., 2013 [21]	Cross-sectional study	PCR	8	PCR	109
			Culture positivity test	8	Culture positivity test	109
			Positive by API smear	8	Positive by API smear	109

EB- Endometrial biopsy, MB- Menstrual blood, PCR- Polymerase Chain Reaction; M-PCR- multiplex PCR, API- Analytical Profile Index, MTD-Gen- Probe Amplified Mycobacterium Tuberculosis Direct Test; AFB- Acid- Fast Bacilli

### Collection of MB

The selected studies used different methods to collect MB. One study used an intrauterine insemination (I.U.I) cannula to collect MB [28], while 3 studies collected MB using a sterile syringe from the cervical cavity [23, 24, 26]. One study obtained a bacteriological culture of MB using a sterile swab [22]. The rest of the studies did not mention the method used to collect MB. Only 5 out of 9 studies mentioned the day MB was collected from the participants [22–24, 26, 27]. Three studies collected MB on the first day of menstruation [22, 24, 27], among which one study mentioned that MB was collected within 12 h of menstruation [22]. And the remaining 2 studies collected MB within two days of menstruation. The number of participants in the MB and EB groups was equal in 4 studies [22, 23, 26, 28]. In all 4 of these studies, MB and EB samples were collected from the same participants. In the remaining studies, there was a huge variation in the number of participants in both groups, with the number of participants in the EB group being more than that of the MB group.

### Type of diagnostic technique used

The diagnostic techniques used are varied across the studies [16, 21–28]. Kamra et al., used M-nested-PCR and M-PCR [27]; Chaubey et al., used nested PCR [26], Paine et al., used PCR and histopathology [23]; Sindhoora et al., used PCR [28]; Patil et al., used VersaTREK™ culture, an automated culture system for the detection of *Mycobacterium tuberculosis*, and Gen-Probe Amplified *Mycobacterium Tuberculosis* Direct Test (MTD), a nucleic acid amplification assay [24] and Malhotra et al., used real-time PCR, smear test and culture positivity test [25]. Kashyap et al., used smear test and culture positivity test [16], Aka et al., used a culture positivity test and histopathology [22] and Singh et al., used PCR, culture positivity test and positive by Analytical Profile Index (API) smear [21].

### Sensitivity and specificity

**Fig. 2:**
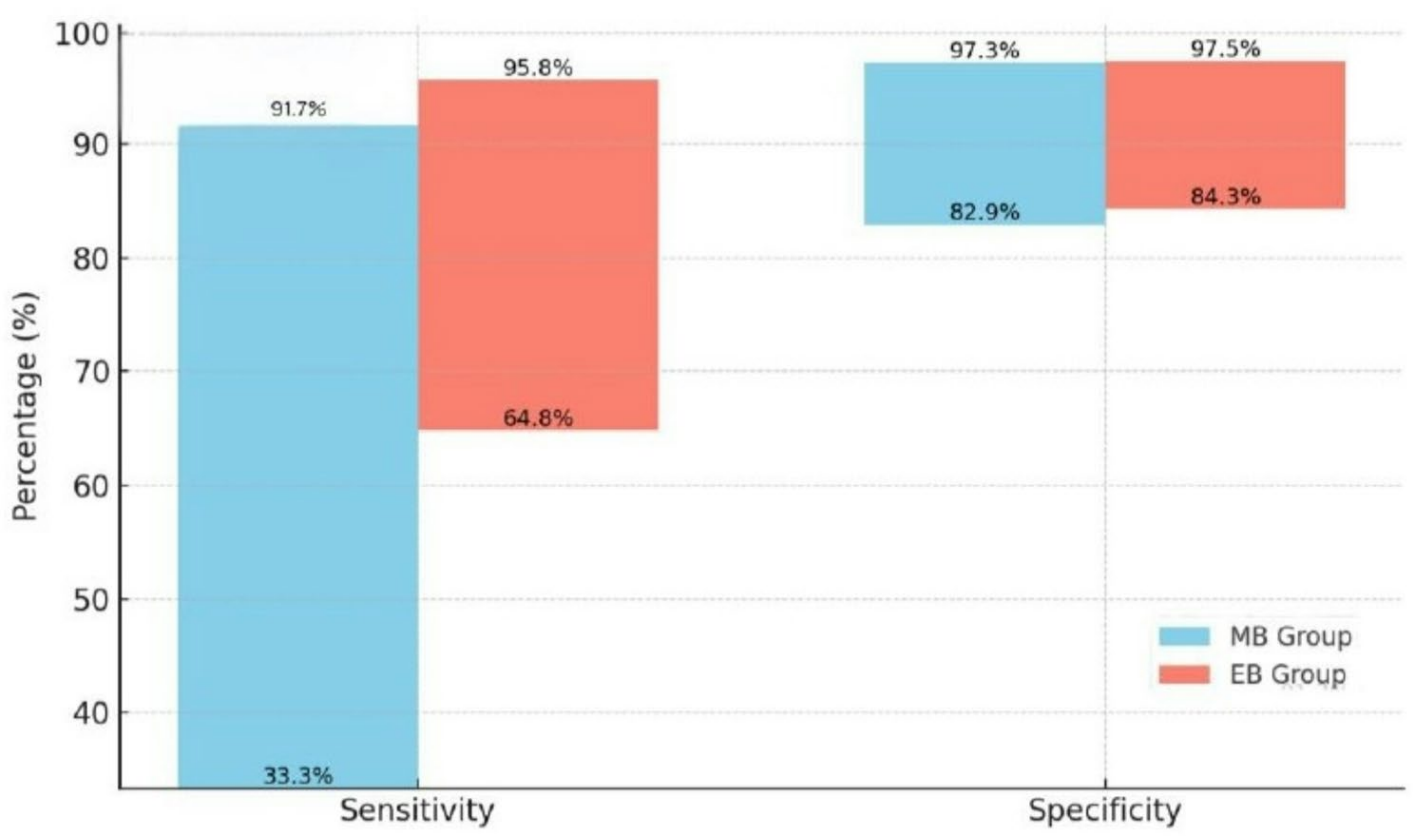
Comparison of the sensitivity and specificity of MB Vs EB samples

Four out of 9 studies measured the sensitivity of various diagnostic techniques in diagnosing FGTB using MB and/or EB (Fig. 2, Table 2). Kamra et al. found that both M-nested PCR and M-PCR showed a sensitivity of 33.3% towards MB [27]. The sensitivity of these M-nested PCR and M-PCR towards EB samples was observed to be 82% and 68.2%, respectively. Similarly, in the study conducted by Chaubey et al., 72% sensitivity was obtained by nested PCR towards MB [26]. In a cross-sectional study by Paine et al., multiplex PCR achieved a sensitivity of 90.2% in MB and 95.80% in EB samples [23]. Similarly, in a cross-sectional study by Sindhoora et al., real-time PCR was able to achieve a sensitivity of 91.67% in both MB and EB samples [28].

**Table 2:** Sensitivity and specificity of included studies

S.N	Study (ref)	Diagnostic test used	Menstrual Blood		Endometrial Biopsy	
			Sensitivity	Specificity	Sensitivity	Specificity
1.	Kamra et al., 2022 [27]	M- nested PCR	33.3%	Not reported	81.8%	97.5%
		M- PCR	33.3%	Not reported	68.2%	92.5%
2.	Chaubey et al., 2019 [26]	nested PCR	72.3%	82.9%	Not reported	Not reported
3.	Paine et al., 2018 [23]	multiplex PCR	90.2%	86.1%	Not reported	Not reported
		PCR	Not reported	Not reported	95.8%	84.3%
		Histopathology	Not reported	Not reported	64.8%	93.2%
4.	Sindhoora et al., 2016 [28]	real-time PCR	91.67%	97.33%	91.67%	97.33%

M-nested PCR- Multiplex Polymerase Chain Reaction; PCR- Polymerase Chain Reaction

In 3 of the 9 studies, the specificity of various diagnostic techniques in diagnosing FGTB using MB and/or EB samples was reported (Table 2). In a cross-sectional study by Chaubey et al., reported that the specificity of nested PCR was 82.90% for MB [26]. Paine et al., found that multiplex PCR achieved a specificity of 86.10% and 84.30% in MB and EB samples, respectively [23]. In the study by Sindhoora et al., PCR achieved a specificity of 97.33% in both MB and EB samples [28].

As indicated in Tables 1 and 2, the included studies used a range of diagnostic tests. For calculating sensitivity and specificity, some studies compared results against culture or histopathological confirmation of *Mycobacterium tuberculosis*, while a few used a composite reference including clinical and molecular findings. 

### Symptoms

Primary infertility was observed to be the highest reported symptom [23–25, 28], followed by secondary infertility [24, 25]. Other symptoms reported were pain in the abdomen [25], abortions [24, 25], menstrual irregularities [23, 24], tube-related complications, pelvic inflammation and endometriosis [23].

### Quality assessment

**Fig. 3:**
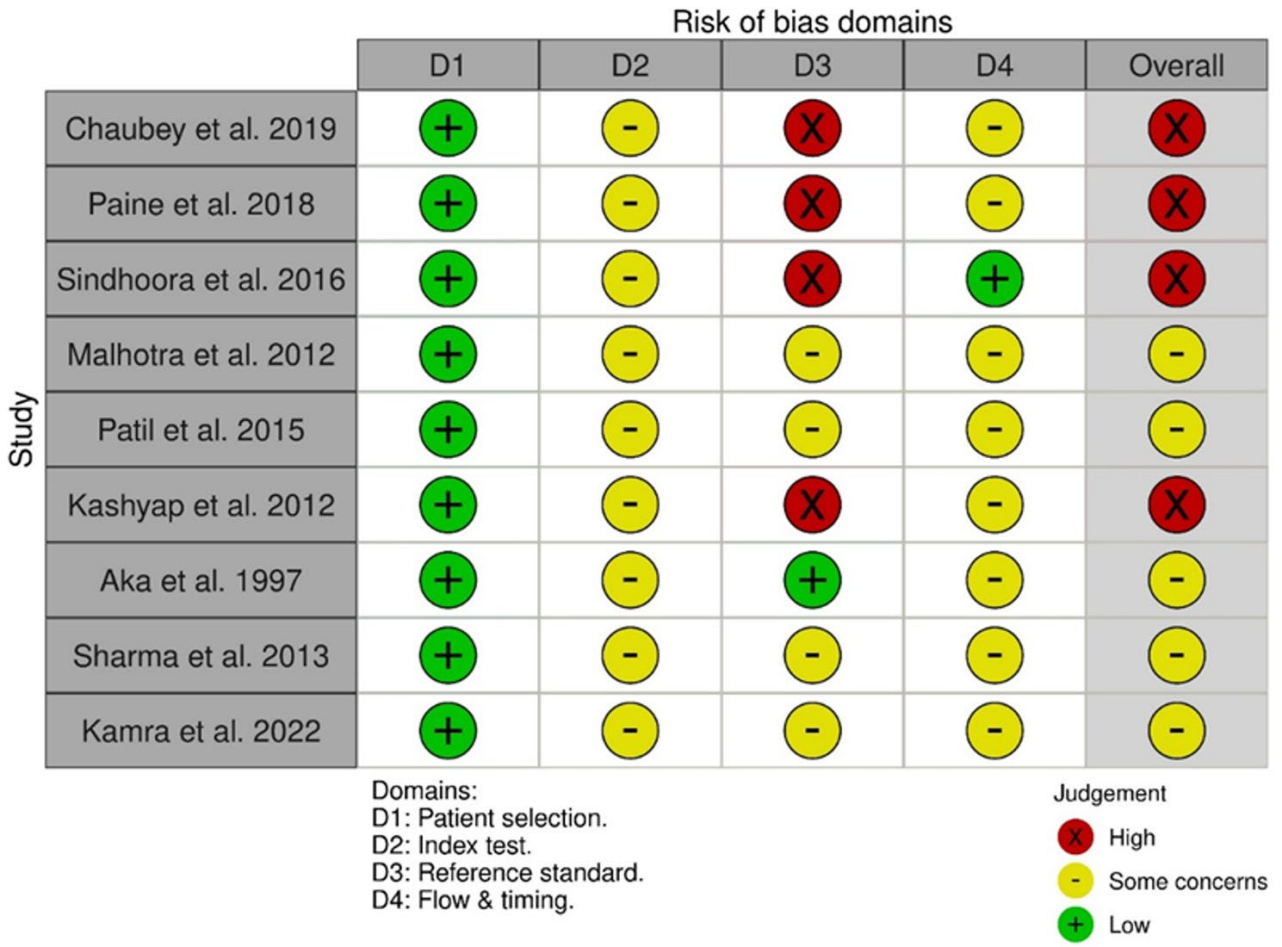
QUADAS-2 Risk of Bias

QUADAS-2 risk of bias assessment scale was employed to assess the methodological quality of the included studies. Figure 3 represent the quality assessment scale of all included studies. Although this review provides a comprehensive qualitative synthesis, a quantitative meta-analysis was not performed because of insufficient or incomplete data on sensitivity and specificity across the included studies. Therefore, none of the studies were excluded to preserve the completeness of the qualitative synthesis.

## Discussion

This systematic review provides new insights into the potential use of MB as a diagnostic specimen for FGTB. While EB remains the established diagnostic standard, its invasive nature often discourages women from undergoing evaluation, especially in low-resource or community-based settings. Across the included studies, MB demonstrated comparable specificity to EB, although sensitivity varied widely depending on the diagnostic technique, bacterial load, and timing of sample collection.

Feasibility and patient comfort are essential in determining the practicality of any diagnostic approach. EB collection requires a specialised procedure that can cause significant discomfort, anxiety, and occasionally post-procedural complications. In contrast, MB collection is simple, non-invasive, and cost-effective. It can be obtained without clinical supervision, making it accessible to women even in remote or resource-limited areas. By reducing procedural discomfort and eliminating the need for specialized facilities, MB sampling could improve patient participation and expand early screening efforts for FGTB.

None of the included studies measured the pain score or the discomfort associated with both methods of sample collection for FGTB diagnosis [16, 21–28]. This highlights a significant gap in assessing participant satisfaction. A questionnaire-based survey to evaluate participant acceptance of MB compared to EB samples would have been very beneficial.

The diagnostic performance of MB-based assays varied across studies, with sensitivity ranging from 33.3% to 91.7% and specificity from 82.9% to 97.3% [23, 26–28]. Despite this variability, MB generally performed well in ruling out FGTB when compared to EB. The reduced sensitivity observed in some studies may reflect lower bacterial loads in MB samples, particularly when infection is localized deeper within the endometrium or fallopian tubes. Furthermore, differences in PCR primer design, target genes, and laboratory methodologies could contribute to this inconsistency. These variations emphasize the need for standardized MB collection procedures and diagnostic protocols to ensure reproducibility and reliability across studies.

The low sensitivity reported in some studies using MB samples may be attributed to several factors. First, the bacterial load in menstrual blood is likely to be lower than in endometrial tissue, especially if the infection is localized deep within the endometrium or fallopian tubes. Second, the timing of sample collection plays an important role, as the concentration of *Mycobacterium tuberculosis* may vary across different days of menstruation. Third, improper collection techniques or contamination can degrade bacterial DNA and affect PCR amplification. This highlights the gap in research scope in understanding how bacterial load concentrations in MB vary across different days of the menstrual cycle. Additionally, differences in diagnostic methodologies (e.g., type of PCR target gene, primer design, and laboratory conditions) and inter-study variability in defining the reference standard could also influence sensitivity. Together, these methodological and biological factors may account for the variation observed in diagnostic performance across studies [16, 21–28].

Infertility is one of the major symptoms and an underdiagnosed factor of FGTB in women. In a meta-analysis of 30,918 infertile women, 20% had FGTB. Among these FGTB patients, the prevalence of overall, primary, and secondary infertility was 88%, 66%, and 34%, respectively [29]. Other symptoms include abdominal pain, amenorrhea, post-menopausal bleeding, menorrhagia, intermenstrual bleeding, infertility, and dyspareunia [7].

The current techniques used to diagnose FGTB in women are the detection of acid-fast bacilli on EB, curettage or aspirate, or histopathology demonstration of epithelioid granuloma on biopsy [30]. However, these are invasive procedures and might lead to severe discomfort and pain, as reported by a prospective study [14], where most women experienced severe pain and high anxiety levels before, during, and after undergoing the EB procedure. Moreover, a significant association was found between pain and anxiety levels, which might impact the number of women willing to undergo a diagnosis of FGTB [14]. It is known that women who are diagnosed and treated early with anti-tubercular drugs have a better chance of spontaneous conception, with rates varying from 31 to 59% [9]. Apart from this, late diagnosis of TB also increases the chances of developing MDR TB [10]. Therefore, opting for an alternative sample that can be obtained using non-invasive techniques is very important. This necessitates strategic interventions that not only focus on non-invasive and cost-effective procedures but also encourage women from all backgrounds to participate in the screening process.

Women of reproductive age typically menstruate for 0–5 days, on average, with 30 mL of blood lost in each cycle. MB is composed of blood from the arteries and veins, prostaglandins, tissue debris and products of fibrinolysis from the endometrial tissue and represents the endometrial environment [31]. Research has also found low intrapersonal variability in the cellular and protein composition of menstrual fluid across each cycle in healthy females [32]. This renders MB a convenient sample for analysis. Apart from this, sanitary pads can be collected and easily stored in a zip-lock bag, unlike EB sample that needs to be stored in a liquid medium [33]. Therefore, MB can produce a reproducible profile and is a readily available sample that can be easily obtained using non-invasive techniques.

MB, usually discarded, has biomarkers similar to those of systemic blood [34]. A cross-sectional study also showed that most of the inflammatory markers were found in higher concentrations in MB than peripheral blood [35]. Currently, studies have shown that MB can be used to diagnose diabetes [19], infectious diseases, and various uterine conditions [36, 37]. In a recent study, tests using MB showed a sensitivity and positive predictive value of 66.7% while detecting human papillomavirus (HPV) [37]. This suggests that MB could be used as a potential sample to detect FGTB. MB for detecting FGTB in women is a practical and convenient approach that provides a more comfortable sample collection than other samples.

Dosnon et al., developed a MenstruAI platform by integrating a wearable sanitary pad with a diagnostic microfluidic system. This was able to semi-quantitatively detect disease and biomarkers like C-reactive protein (CRP), endometriosis biomarker CA-125, and cancer biomarkers (CEA and CA-125). This platform could change its colour in the presence of biomarkers, which could be easily seen through the naked eye, as well as read through machine learning algorithms [38]. Similarly, in a study by Naseri et al., it was observed that MB collected on a pad showed high concordance with clinically collected cervical specimens among women who tested positive for HPV [39]. Wang et al., successfully developed a microfluidic chip using hemagglutinin antibodies to capture the *Mycobacterium tuberculosis* bacteria; this chip was able to automate the entire bacterial detection process within 90 min [40]. Integration of such chips into the sanitary pads would make the entire diagnostic process of FGTB much more convenient and simpler.

Additionally, utilizing MB might encourage more women to undergo screening for FGTB. In a questionnaire-based study by Wong et al., women were asked to choose between MB and Papanicolaou test (Pap) smear tests to obtain a sample and diagnose a disease [33]. It was observed that around 87% of women opted for an MB sample rather than a Pap smear test. Not only can this sample be obtained comfortably, but it is also less time-consuming. As most of the rural women are daily wage workers, collecting MB will not affect their daily routine or wage-earning potential, and might also improve the number of women willing to undergo the screening [41]. Additionally, MB can be used to check disease prognosis and the progress of treatment as the menstrual cycle occurs every month. However, this may not be possible for women with irregular menstrual cycles, which is a commonly observed symptom in females with FGTB [2].

From a clinical perspective, MB-based testing may serve as a feasible preliminary screening or triage tool for reproductive-age women suspected of FGTB. Integrating MB testing as an initial step could reduce reliance on immediate invasive procedures, thereby improving patient comfort and healthcare accessibility. A stepwise diagnostic model where MB-positive results prompt confirmatory EB testing could improve early case detection, reduce diagnostic delays, and optimize use of limited medical resources. This model aligns with public health goals of increasing screening uptake while minimizing patient discomfort.

However, MB sampling is not universally applicable. Amenorrheic women, postmenopausal patients, or those with irregular menstrual cycles cannot provide MB samples. In these cases, EB or alternative specimens, such as endocervical swabs or peritoneal aspirates, remain essential for accurate diagnosis. Therefore, MB should be viewed as a complementary diagnostic specimen rather than a replacement for EB within clinical workflows.

The current review has a few limitations. The methodological diversity among the included studies also introduces potential bias. Among the available studies, variability in diagnostic techniques, differences in sample sizes between the MB and EB groups, and the limited availability of sensitivity and specificity data (reported in only four of the nine studies) made it difficult to extract sufficient information for performing a meta-analysis. Consequently, the diagnostic accuracy of MB versus EB relies heavily on a small subset of primary studies, which restricts the generalizability of the findings. Furthermore, most of the included studies were conducted in India, with only one reported from Turkey, which may introduce regional bias and limit the external validity of the results. Hence, future research should include larger, multicentric studies across diverse populations to better establish the diagnostic reliability of menstrual blood in detecting FGTB.

Although this review supports the feasibility and potential utility of MB-based testing, further research is necessary. Future studies should standardize MB collection and testing methodologies, assess bacterial load variations across menstrual phases, and evaluate diagnostic performance using uniform reference standards. Moreover, patient-centered studies exploring pain perception, satisfaction, and acceptance of MB sampling would provide valuable insights into its implementation in real-world settings.

## Conclusion

This systematic review highlights that MB demonstrates promising diagnostic potential for detecting FGTB, offering a non-invasive, patient-friendly, and accessible alternative to EB. Across the included studies, the specificity of MB-based diagnostic methods was generally comparable to that of EB, although sensitivity varied widely depending on factors such as bacterial load, timing of collection, and diagnostic technique. These findings suggest that MB could serve as a feasible screening or adjunct specimen in clinical settings, particularly in low-resource regions or among women who are reluctant to undergo invasive procedures.

While these findings support the potential utility of MB as an adjunct or preliminary screening tool, they do not yet justify replacing EB in clinical practice. MB collection may not be possible in certain groups, such as amenorrheic women or those with irregular menstrual cycles, and current data are geographically limited. Therefore, larger, multicentric studies are needed to confirm these findings, refine diagnostic protocols, and evaluate the integration of MB-based testing into clinical workflows for FGTB detection.

## Supplementary Information

Below is the link to the electronic supplementary material.

Supplementary Material 1

Supplementary Material 2

## Data Availability

All data generated or analysed during this study are included in this published article [and its supplementary information files].

## Abbreviations

CRP: C-reactive protein
EB: Endometrial biopsy
EPTB: Extrapulmonary tuberculosis
FGTB: Female genital tuberculosis
IUI: Intrauterine insemination
MB: Menstrual blood
MDR: Multi-drug resistant
MeSH: Medical Subject Headings
M-PCR: PCR-Multiplex Polymerase Chain Reaction
PCR: Polymerase Chain Reaction
PRISMA: Preferred reporting items for systematic review and meta-analysis
PROSPERO: Prospective register of systematic reviews
QUADAS-2: Quality Assessment of Diagnostic Accuracy Studies-2
