# Supplementary material for: Comparison of menstrual blood and endometrial biopsy as specimens in the diagnosis of female genital tuberculosis: a systematic review
Source: BMC Infect Dis. 2026 Jan 29;26:436. doi: 10.1186/s12879-026-12564-8 (PMC12924429; doi:10.1186/s12879-026-12564-8)
Supplement: Supplementary file 1 — Supplementary Material 1 [file 12879_2026_12564_MOESM1_ESM.docx]

**SCOPUS** – 7/03/2024 (filters- title, abstract and keywords) = 67 results

 "Menstrual blood samples" OR "Menstrual blood"

AND

"genital tuberculosis" OR "Female genital tuberculosis" OR "Mycobacterium tuberculosis" OR "tuberculosis"

**EMBASE-** 7/03/2024 (filters- title, abstract and keywords) = 47 results

 ‘Menstrual blood samples’ OR ‘Menstrual blood’

AND

‘genital tuberculosis’ OR ‘Female genital tuberculosis’ OR ‘Mycobacterium tuberculosis’ OR ‘tuberculosis’

**WEB of SCIENCE** – 7/03/2024 ( filters- title) = 4 results

Menstrual blood samples OR Menstrual blood

AND

genital tuberculosis OR Female genital tuberculosis OR Mycobacterium tuberculosis OR tuberculosis

**PUBMED –** 7/03/2024 ( filters- no filters)= 115 results

((Menstrual blood samples) OR (Menstrual blood)) AND ((((genital tuberculosis) OR (Female genital tuberculosis)) OR (Mycobacterium tuberculosis)) OR (tuberculosis))
